# Radiological markers of neurological manifestations of post-acute sequelae of SARS-CoV-2 infection: a mini-review

**DOI:** 10.3389/fneur.2023.1233079

**Published:** 2023-11-24

**Authors:** Olivia Cull, Lina Al Qadi, Josiane Stadler, Mykella Martin, Antonios El Helou, Jeffrey Wagner, Danica Maillet, Ludivine Chamard-Witkowski

**Affiliations:** ^1^Centre de formation médicale du Nouveau Brunswick, University of Sherbrooke, Moncton, NB, Canada; ^2^New Brunswick Center for Precision Medicine, Moncton, NB, Canada; ^3^Faculty of Medicine, Dalhousie University, Halifax Regional Municipality, Halifax, NS, Canada; ^4^Faculty of Medicine, Memorial University, St John's, NL, Canada; ^5^Department of Neurosurgery, The Moncton Hospital, Moncton, NB, Canada; ^6^Department of Diagnostic Imaging, The Moncton Hospital, Moncton, NB, Canada; ^7^Vitalité Health Network, Dr. Georges-L.-Dumont University Hospital Centre, Moncton, NB, Canada; ^8^Department of Neurology, Dr.-Georges-L.-Dumont University Hospital Center, Moncton, NB, Canada

**Keywords:** Neuro-PASC, post-COVID, MRI, fMRI, PET/CT scan, imaging, SPECT (single-photon emission computed tomography)

## Abstract

The neurological impact of COVID-19 is a rising concern among medical professionals, as patients continue to experience symptoms long after their recovery. This condition, known as neurological post-acute sequelae of COVID-19 (Neuro-PASC), can last for more than 12 weeks and includes symptoms such as attention disorders, brain fog, fatigue, and memory loss. However, researchers and health professionals face significant challenges in understanding how COVID-19 affects the brain, limiting the development of effective prevention and treatment strategies. In this mini-review, we provide readers with up-to-date information on the imaging techniques currently available for measuring the neurological impact of post-SARS-CoV-2 infection. Our search of PubMed and Google Scholar databases yielded 38 articles on various brain imaging techniques, including structural MRI (magnetic resonance imaging), functional MRI, diffusion MRI, susceptibility-weighted imaging, SPECT (single-photon emission computed tomography) imaging, and PET (positron emission tomography) imaging. We also discuss the optimal usage, limitations, and potential benefits of these techniques. Our findings show that various cerebral imaging techniques have been evaluated to identify a reliable marker for Neuro-PASC. For instance, ^18^F-FDG-PET/CT and functional MRI have demonstrated hypometabolism in cerebral regions that are directly linked to patient symptoms. Structural MRI studies have revealed different findings, such as infarcts, white matter atrophy, and changes in gray matter volumes. One SPECT imaging study noted frontal lobe hypometabolism, while diffusion MRI showed increased diffusivity in the limbic and olfactory cortical systems. The sequence SWI showed abnormalities primarily in white matter near the gray-white matter junction. A study on ^18^F-amyloid PET/CT found amyloid lesions in frontal and anterior cingulate cortex areas, and a study on arterial spin labeling (ASL) found hypoperfusion primarily in the frontal lobe. While accessibility and cost limit the widespread use of ^18^F-FDG-PET/CT scans and functional MRI, they seem to be the most promising techniques. SPECT, SWI sequence, and ^18^F-amyloid PET/CT require further investigation. Nevertheless, imaging remains a reliable tool for diagnosing Neuro-PASC and monitoring recovery.

## 1 Introduction

Since making its debut in December of 2019, COVID-19 (SARS-CoV-2) has infected nearly 750 million people around the globe, according to the World Health Organization (1). Primarily presenting as a respiratory syndrome, many have reported neurological manifestations, termed “neuro-COVID” either in the acute setting of the disease, or neurological symptoms lingering far after respiratory recovery (2). Approximately one-third of the population affected with COVID-19 and nearly two-thirds of hospitalized COVID-19 patients have experienced neurological complications from their infection (3, 4). Common acute neurological complications, lasting <4 weeks, include anosmia, dysgeusia, altered mental status, encephalopathy, peripheral neuropathy and acute cerebrovascular events (3, 4). On the other hand, nearly 10% of people affected by COVID-19 have signs or symptoms that develop during or after COVID-19 and that persist for over 3 months (5). This was classified as post-COVID syndrome. There is some discrepancy with regard to the accepted time frame to be classified as “post-COVID”, ranging between 4 weeks and 12 weeks after the acute phase (6). To further distinguish the acute from the persistent or chronic post-COVID syndrome, the neurological symptoms associated with long-term post-COVID syndrome are different than those present in the acute phase, and they are referred to as Neuro PASC (Neurological manifestations of Post-Acute Sequelae of SARS-CoV-2 infection (7). Fatigue, memory loss, brain fog, anosmia, attentional disorders, subjective cognitive impairment, and headaches dominate the clinical board when it comes to prolonged coronavirus implications (4, 8). These have been seen to persist for over 6 months following onset (5).

### 1.1 Pathophysiology of neuro-COVID

Three main hypotheses are currently considered to explain Neuro-PASC (8). The first is by indirect mechanism, via peripheral inflammation, also known as the “cytokine storm” that is triggered by SARS-CoV-2. This systemic inflammatory response could alter the equilibrium of the brain, similar to a septic encephalopathy (9). Cytokine storms occur when the fine balance between pro-inflammatory and anti-inflammatory cytokines is lost, and a previously localized inflammation affects the entire system. This deregulation can be non-provoked, as in auto-inflammatory illnesses, or can be precipitated by an infectious agent, as is the case for COVID-19. In the latter, studies have shown an elevation of IL-1β, IL-2, IL-6, IL-10, IFN-γ, and TNF-α, amongst others, and that the degree of elevation was proportional to the severity of the illness (10). The second theory is also one of indirect mechanism, and rests on the basis of lack of perfusion, sepsis or hyperpyrexia that can accompany the respiratory infection, each with their respective toll on the brain's homeostasis. The third hypothesis is one of direct neurotropism (11). SARS-CoV-2 infects the human body by binding to ACE2 receptors, which are primarily found in type 2 alveoli of the lungs (12) and entering the cells. Particularly, ACE2 receptors are also present in the brain's vascular endothelium and smooth muscles, as well as in skeletal muscles, thus explaining why myalgia is a widely present symptom included in post-COVID syndrome. Skeletal muscle cells express ACE2 receptors, which makes them a direct target for SARS-CoV-2 invasion (8). In support of this third theory, previous studies demonstrated that SARS-CoV-2 enters the nervous system by latching onto ACE2 receptors of the olfactory mucosa, penetrating the neural-mucosal interface, the only part of the brain not protected by the dura (3), and migrating along neuronal structures, eventually leading to the centers controlling cardiorespiratory functions (11). Whether Neuro-PASC is caused by direct attack of the virus on the brain or indirectly from systemic secondary damages, or by a combination of these (2), needs to be clarified.

### 1.2 The need for a precise marker

Despite the immensely rapid response and development of COVID-19 management protocols, many gaps need to be filled when it comes to understanding the pathophysiology of the disease as a whole. For example, up to 55% of hospitalized COVID-19 patients have reported to have neurological manifestations 3 months after their infection (2). This is still not well investigated and studied. This article serves as a review of the radiological markers that are currently in development for the evaluation of neurological damages of COVID-19. We will discuss ^18^F-FDG-PET/CT, SPECT, and ^18^F-AMYLOID PET/CT imaging, as well as structural, functional, diffusion MRI, ASL, and susceptibility-weighted imaging, and their current contribution in the management of COVID-19 specifically from a neurological standpoint.

## 2 Methods

### 2.1 Search strategy

A record search was performed to identify neuro-imaging methods for tracing Neuro-PASC including ^18^F-FDG-PET/CT, SPECT, MRI and fMRI. Databases searched were PubMed and Google Scholars for papers published between March 2020 and April 2023. Searches were divided by neuro-imaging methods for tracing Neuro-PASC. Studies were included if they (1) examined neurological markers, (2) used neuro-imaging modalities, (3) studied patients with a COVID-19 history and, (4) were in English. We excluded articles that examined children, adolescents and pregnant women or consisted of post-vaccination studies.

### 2.2 Search chains

With regards to 18 F-FDG-PET/CT imaging, the search key was ((((Neuro^*^) OR (brain)) AND ((Covid^*^) OR (coronavirus) OR (SARS-CoV-2))) AND ((long covid) OR (post covid))) AND ((PET) OR (positron)). For SPECT imaging, ((((Neuro^*^) OR (brain)) AND ((Covid^*^) OR (coronavirus) OR (SARS-CoV-2))) AND ((long covid) OR (post covid))) AND ((SPECT) OR (single photon)). For 18F-amyloid PET/CT, ((((Neuro^*^) OR (brain)) AND ((Covid^*^) OR (coronavirus) OR (SARS-CoV-2))) AND ((long covid) OR (post covid))) AND ((amyloid) OR (amyloid PET)). For structural MRI, (((((Neuro^*^) OR (brain)) AND ((Covid^*^) OR (coronavirus) OR (SARS-CoV-2))) AND ((long covid) OR (post covid))) AND ((MRI) OR (Magnetic resonance imaging)). For functional MRI, ((((Neuro^*^) OR (brain)) AND ((Covid^*^) OR (coronavirus) OR (SARS-CoV-2))) AND ((long covid) OR (post covid))) AND ((fMRI) OR (functional MRI)) yielded no results addressing our topic's subject. For ASL MRI, ((((Neuro^*^) OR (brain)) AND ((Covid^*^) OR (coronavirus) OR (SARS-CoV-2))) AND ((long covid) OR (post covid))) AND ((ASL MRI) OR (arterial spin labeling)). For diffusion MRI, ((((Neuro^*^) OR (brain)) AND ((Covid^*^) OR (coronavirus) OR (SARS-CoV-2))) AND ((long covid) OR (post covid))) AND ((diffusion MRI) OR (dMRI)). For susceptibility-weighted imaging, ((((Neuro^*^) OR (brain)) AND ((Covid^*^) OR (coronavirus) OR (SARS-CoV-2))) AND ((long covid) OR (post covid))) AND ((SWI) OR (Susceptibility-weighted imaging)). A targeted search was done on Google Scholar and PubMed for each neuro-imaging modality and post-covid (i.e.: post-covid PET neuro finding) to identify specific articles that may have been omitted.

### 2.3 Selection strategy

**Figure 1:**
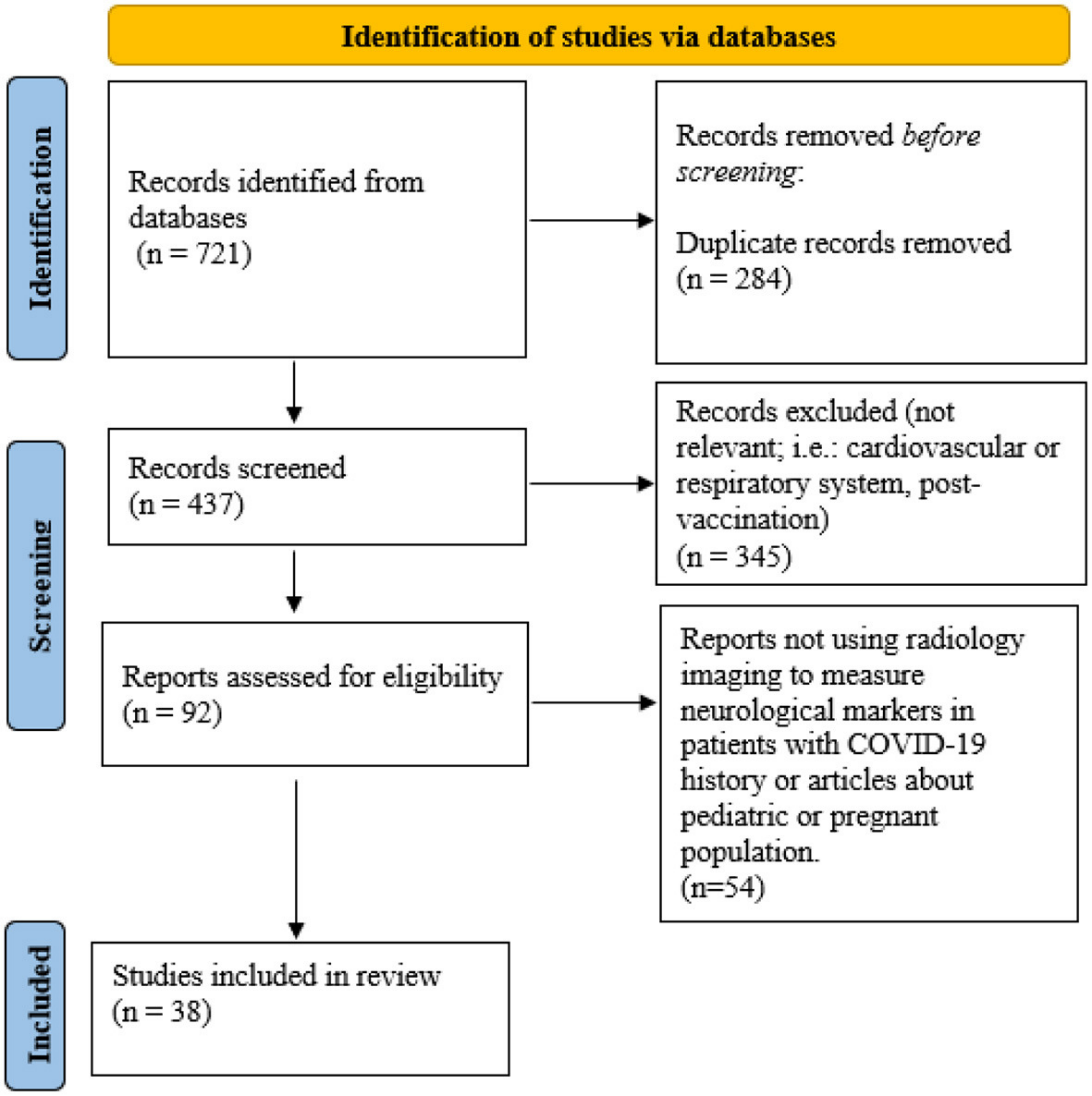
Prisma flowchart.

Retained articles were checked for duplicates and were selected after undergoing a title, abstract and eventually a full text screening. The screening and selection process was done by two independent authors (OC and MM). Collectively, 92 abstracts were read and 38 were used to construct this review. Specifically, 1 for SPECT imaging, 9 for advances with ^18^F-FDG-PET/CT, 1 for ^18^F-Amyloid-PET/CT, 10 for structural MRI, 2 for functional MRI, 1 for diffusion MRI, 2 for SWI and the remainder for additional information on Neuro-PASC. A PRISMA flowchart can be found in Figure 1.

## 3 Results

For each selected article, the following data was extracted and summarized in Table 1: author, journal, study design, number of subjects, number of controls, gender distribution, age, time elapsed since COVID-19 infection, type of imaging and main findings.

**Table 1:** Summary of the characteristics and findings of the imaging studies included in this review.

**Authors**	**Journal**	**Study design**	**Number of subjects**	**Number of controls**	**Gender distribution male/female**	**Age (years)**	**Time since COVID-19 infection**	**Type of imaging**	**Main findings in patients**
Sollini et al. (13)	Springer Nature	Prospective case-control study	13	26	8/5	54 (46–80)	132 ± 31 days	^18^F-FDG PET/CT	^18^F-FDG uptake in “target” and “non-target” tissues. Relative hypometabolism in the right parahippocampus and thalamus. Specific region(s) of hypometabolism in patients with persistent anosmia/ageusia, fatigue, and vascular uptake.
Karimi-Galougahi et al. (14)	Academic Radiology	Case study	1	N/A	0/1	27	6 weeks	^18^F-FDG-PET/CT	Hypometabolism in the orbitofrontal cortex.
Donegani et al. (15)	Biomedicines	Longitudinal cross-sectional cohort study	22	61	12/10	64 ± 10.5	>1 month	^18^F-FDG-PET/CT	Relative hypometabolism shown in bilateral parahippocampus and fusiform gyri and in left insula.
Hosp et al. (9)	Oxford University Press	Prospective cohort study	29	45	18/11	65.2 ± 14.4	1 month average	3T sMRI and ^18^F-FDG PET/CT	MRI showed subacute infarcts. ^18^F-FDG PET/CT showed predominant frontoparietal hypometabolism.
Guedj et al. (16)	Springer Nature	Retrospective case control study	35	44	20/15	55.06 ± 11.22	≥3 weeks	^18^F-FDG PET/CT	Hypometabolism in olfactory gyrus and connected paralimbic/limbic regions, extended to the cerebellum and brainstem.
Dressing et al. (17)	The Journal of Nuclear Medicine	Prospective cohort study	14	45	5/9	56.3 ± 7.2	198.0 ± 63.5 days	^18^F-FDG PET/CT	No pathological findings found on imaging.
Debs et al. (18)	American Journal of Neuroradiology	Retrospective single-center study	45	52	24/21	58 (18–87)	6.57 ± 4.85 months	^18^F-FDG-PET/CT	Focal hypometabolism peak in cerebellum and in bilateral frontal, parietal, occipital, and posterior temporal lobes, during the first 2 months, nearly resolved at 6 months and disappeared at 12 months. Hypermetabolism in brainstem, cerebellum, limbic structures, frontal cortex, and periventricular white matter shown 2–6 months post-infection.
Jamoulle et al. (19)	Viruses	Mixed method, cohort study (Action research)	55 (32 for imaging)	N/A	15/40	42.4 (12–79)	13.3 ± 8.9 (recovered mild and severe) 18.3 ± 5.9 (still ill, very severe)	SPECT-CT	SPECT-CT showed cerebral hypoperfusion lesions consistent with the severity of the condition.
Ferrucci et al. (20)	Springer Nature	Retrospective case study	7	N/A	4/3	56 ± 12.39	>12 months	^18^F-FDG PET/CT and ^18^F-amyloid PET/CT	^18^F-FDG PET/CT showed various hypometabolism in the left temporal mesial, pontine, and bilateral prefrontal and parietal regions. ^18^F-amyloid PET/CT done for the patient with the greatest extent of hypometabolism showed significant Aβ deposition in the superior and middle frontal cortex, in the posterior cingulate, and mildly in the rostral and caudal anterior cingulate regions.
Thapaliya et al. (21)	Frontiers in Neuroscience	Cross-sectional study	8	10	3/5	43.2 ±10.7	≥3 months	7T sMRI	Strong negative relationship between midbrain volume and “breathing difficulty”. Greater pons and whole brainstem volumes in patients.
Cecchetti et al. (22)	Springer Nature	Longitudinal quasi-experimental study	36	36	25/11	58.5 ± 13.3	≥30 ± 15 days	3T sMRI	No significant differences in total brain, gray, or white matter volumes were found between patients and controls. Greater total of right frontal and right parieto-occipital white matter hyperintensity volumes. Interrelated cognitive, EEG and MRI alterations were observed after two months of COVID-19 resolution.
Douaud et al. (23)	Nature	Longitudinal quasi-experimental cohort study	401	384	172/229	62.1 ± 6.7	141 ± 79 days	sMRI, fMRI, dMRI	Reduced total brain size, gray matter thickness and tissue contrast in orbitofrontal cortex and parahippocampal gyrus. Greater changes in patients regarding diffusion measures in areas functionally connected to the primary olfactory cortex.
Kandemirli et al. (24)	Academic Radiology	Prospective case study	23	N/A	9/14	29 (22–41)	1–4 months	Paranasal sinus CT and 3T MRI dedicated to olfactory nerves	CT showed opacification in olfactory cleft. MRI showed olfactory bulb degeneration and changes in shape. MRI also showed diffused increase in signal intensity from the olfactory bulb, scattered hyperintense foci, and microhemorrhages.
Besteher et al. (25)	Psychiatry Research	Cross-sectional study	30	20	13/17	47.5 ± 11.5	8.65 (2–16) months	3T sMRI	Multiple clusters of significant bilateral gray matter volume enlargement in fronto-temporal regions, insula, hippocampus, amygdala, basal ganglia, and thalamus.
Hellgren et al. (26)	BMJ Open	Ambidirectional observational cohort study	35	N/A	28/7	59 (51–66)	7 months	3T SWI, DWI, and sMRI	Multiple findings of white matter lesions near gray-white matter junction in frontal and parietal lobes.
Campabadal et al. (27)	Annals of Clinical and Translational Neurology	Prospective cohort study	48	N/A	10/38	48.04 ± 7.5 (normal olfaction) 51.96 ± 7.92 (olfactory dysfunction)	9.94 ± 3.83 months	3T sMRI and DWI	Reduced gray matter volume and increased mean diffusivity in olfactory-related areas explaining persistent olfactory deficits in patients. Greater radial diffusivity in the anterior corona radiata, the genu of the corpus callosum, and uncinate fasciculus in patients with deficits compared to those without.
Yus et al. (28)	Acta Neurologica Scandinavica	Cross-sectional study	82	N/A	24/58	51.74 ± 10.85	11.18 ± 3.78 months	ASL, sMRI, and DTI	Olfactory dysfunction associated with lower tissue perfusion in orbital and medial frontal areas. Absence of statistically significant findings in brain volumes and diffusion-tensor imaging.
Kiatkittikul et al. (29)	Nuclear Medicine and Molecular Imaging	Retrospective case study	13	N/A	6/7	47 (42–54)	>28 days	^18^F-FDG PET/CT and 3 T PET/rsfMRI	^18^F-FDG PET/CT showed uptake in many organs. ^18^F FDG PET showed many areas of hypometabolism in the thalamus, and in parietal, temporal, frontal, and occipital lobes. rsfMRI results showed abnormal brain connectivity which is coherent with ^18^F-FDG PET findings.
Churchill et al. (30)	Frontiers in Neurology	Cross-sectional study	51	15	17/34	41 ± 12	4–5 months average	3 T sMRI and fMRI	Lower connections in the thalamus and decreased temporal subcortical functional connectivity in patients.
Ajčević et al. (31)	Nature Portfolio	Prospective cohort study	24	22	9/15	53.0 (±14.5)	≥4 weeks	ASL MRI	Hypoperfusion predominantly in frontal, parietal, and temporal cortex.
Mishra et al. (32)	medRxiv pre-print, not peer reviewed	Prospective cohort group-level study	46	30	31/15	34.67 ± 9.51	< 6 months	3T SWI and sMRI	Higher susceptibility imaging values in the frontal lobe and brainstem in patients. Observed cluster in midbrain and bilateral clusters in white matter near the orbitofrontal gyri and the gray-white matter junctions. Significant clusters negatively correlated with fatigue scores found in frontal lobe, anterior cingulate cortex, and brainstem.

^18^F-FDG PET/CT, Positron emission tomography with 18 fluoro-D-glucose integrated with computed tomography; SPECT-CT, Single Photon Emission Tomography-Computed Tomography; sMRI, structural MRI; dMRI, diffusion MRI; SWI, susceptibility-weighted imaging; DWI, diffusion-weighted imaging; DTI, diffusion-tensor imaging; ASL, arterial spin labeling.

### 3.1 ^18^F-FDG-PET/CT

Positron emission tomography (PET) measures the emission of positrons from radiomarked tracer molecules, allowing localization of metabolically active processes. The most widely used radiotracer is currently ^18^F-Fluorodeoxyglucose (^18^F-FDG), a radiolabeled glucose molecule. This method allows localization of regions of abnormal increase or decrease glucose intake in the body to better identify hyper or hypometabolic states and is currently mainly used as part of the workup and follow-up in oncology. In the Neuro-PASC scope, the expected utility of ^18^F-FDG-PET/CT is to identify a specific cerebral target that explains the clinical manifestations (33). This will be useful to better determine the pathophysiology, to improve the accuracy of the diagnosis, to then better develop therapeutic options and accurate follow-ups. When the intrinsic role of the hyper/hypometabolic brain loci correlates with the clinical manifestations of Neuro-PASC, while it cannot prove causality, points toward understanding the pathophysiology of COVID-19 and helps clinicians make the diagnosis of Neuro-PASC.

Sollini et al.'s case control study in 2021 evaluated post-COVID syndrome hallmarks on ^18^F-FDG-PET/CT and showed brain hypometabolism in limbic, paralimbic, brainstem and cerebellum. In addition, none of the post-COVID patients exhibited regions of hypermetabolism compared to controls. Limbic and orbito-frontal hypometabolism correlated with the anosmia present during acute COVID-19 infection. Sollini's team also evaluated the whole-body ^18^F-FDG-PET/CTscans. Despite the hypometabolic activity in the nervous system, they found hypermetabolic activity in different organs, independent of ACE-2 receptor activity, in other words, regions of hypermetabolism were seen in target (ACE-2 presenting) as well as non-target organs, supporting the hypothesis that systemic inflammation may be in play (13).

Verger et al.'s (34) study showed that 47% of the ^18^F-FDG-PET/CT scans of 143 patients with suspected Neuro-PASC had hypometabolism in limbic/paralimbic and fronto-orbital olfactory regions, as well as in the brainstem and cerebellum.

Several other studies have been conducted using ^18^F-FDG-PET/CT imaging to reveal potential Neuro-PASC biomarkers. A study by Karimi-Galougahi et al. (14) brought forth reduced metabolic activity in the orbitofrontal cortex using ^18^F-FDG-PET/CT. Bilateral par hippocampal, fusiform gyri, and left insula hypometabolism were seen in an ^18^F-FDG-PET/CT imaging study conducted by Donegani et al. (15).

A study by Hosp et al. (9) that ran its course in parallel with the Montreal Cognitive Assessment test (MoCA), a highly sensitive tool for early detection of mild cognitive impairment (MCI), correlated cognitive decline with the evident frontoparietal hypometabolism seen on scan. This study showed that while a structural MRI could not find any sign of cerebral damage, ^18^F-FDG-PET/CT discovered cortical fronto-parietal hypometabolism.

A study by Guedj et al. (16), demonstrated that clusters of patients with hypometabolic patterns on ^18^F-FDG-PET/CT scans, flagged by whole-brain statistical analysis performed using SPM8, were very distinct in patients with Neuro-PASC and allowed to reliably differentiate their brain from one of a control subject. The findings suggested that hypometabolism was seen in bilateral rectal/orbital gyrus, including the olfactory gyrus, right temporal lobe, bilateral pons/medulla, and the cerebellum bilaterally. These patients exhibited many functional complaints. Patients with cerebellar hypometabolism experienced hyposmia, anosmia and memory impairment. Patients with frontal cortex, brainstem and cerebellum hypometabolism presented pain and insomnia.

^18^F-FDG-PET/CT has also proven to be useful in characterizing the timeline of damages caused by SARS-CoV-2. Post-COVID syndrome patients who had increased vascular uptake (hypermetabolism) on whole-body scan done at 1-month post-infection also showed hypometabolism in the brain, thus demonstrating the different effects of COVID-19 on different regions of the body, and the chronological sequence for brain and whole-body changes throughout the disease (13).

In contrast to these studies, Dressing et al.'s (17) study revealed that post-COVID patients reporting symptoms lasting for over 3 months after the acute infection, actually only presented mild impairment on cognitive testing (MoCA) with their distinct pathologic findings on ^18^F-FDG-PET/CT. In this same stretch of ideas, Ferruci et al.'s review explained that not all patients who presented cognitive decline in the post-COVID timeframe showed hypometabolism on ^18^F-FDG-PET/CT (20).

In Debs et al. (18) study, a time-dependent brain PET hypo- and hypermetabolism was observed in patients with a history of COVID-19. Hypometabolic activity, in the bilateral frontal, parietal, occipital, and posterior temporal lobes and cerebellum, reached its peak at 2 months post-infection, then nearly resolved at 6 months, followed by disappearance at 12 months. Additionally, 2–6 months after infection onset, hypermetabolism was observed in brainstem, cerebellum, limbic structures, frontal cortex, and periventricular white matter.

### 3.2 SPECT/CT

Single Photon Emission Computed Tomography (SPECT/CT) is a method that allows superposition of anatomical images with metabolic activity. While PET scans detect metabolism through emission of photons, SPECT scans detect gamma rays from tracers injected into the patient (35). Brain SPECT measures brain perfusion. A limited number of studies met the particular interest of this topic. Takao et al.'s (36) study on Neuro-PASC patients described observing hypoperfusion on SPECT imaging in various areas of the brain, particularly the frontal lobes, which correlates with the previous findings in 18 F-FDG-PET/CT scans. A study conducted on 32 patients with highly impaired functional status, determined by COOP/WONCA functional ability questionnaire (Cooperative Research Network/World Organization of National Colleges, Academies and Academic Associations of General Practitioners/Family Physicians), demonstrated cerebral perfusion changes on SPECT/CT in 29 patients (90%) (19). Fifteen patients then underwent a control SPECT-CT 3–9 months after their initial imaging taken during the acute infection. The follow-up image showed marked improvement in 8 cases and worsening in 7 cases.

### 3.3 ^18^F-AMYLOID PET/CT

Amyloid PET is an imaging modality that allows the visualization of amyloid plaques. A retrospective study conducted by Ferrucci et al. (20) found amyloid plaques in one Neuro-PASC patient 12 months after hospital discharge for his acute COVID-19 infection using an ^18^F-amyloid PET/CT. The plaques were found in the superior and middle frontal cortex, in the posterior cingulate cortex and in the rostral and caudal areas of the anterior cingulate cortex in a lower quantity. ^18^F- FDG PET/CT also detected hypometabolism in the left mesial temporal cortex of this patient (20). To our knowledge, this is the only published article that examined Amyloid PET.

### 3.4 Structural MRI

The anatomy of the brain can be studied using an MRI, an imaging modality that allows the visualization of soft tissues using a magnetic field (37). Results by Hosp et al. (9) suggest that ^**18**^F-FDG-PET/CT seems to be more effective in detecting anomalies than 3T MRI. Specifically, ^**18**^F-FDG-PET/was able to detect frontoparietal hypometabolism, whereas 3T MRI found no relevant structural or vascular anomalies. The sequences used in this article were as follow: sagittal 3D-T1 rapid gradient echo (MP-RAGE) before and after contrast infusion, sagittal 3D FLAIR SPACE (sampling perfection with application-optimized contrasts using different flip angle evolutions), SWI and diffusion mesoscopic imaging (DMI). Nonetheless, 3T MRI allowed to localize a few subacute infarcts (9).

However, in a pilot study conducted by Thapaliya et al. (21), T_1_-weighted 7T MRI showed an increased volume of the superior cerebella peduncle, pons, and entire brainstem in Neuro-PASC patients compared to controls. These biomarkers were also found in patients with myalgic encephalomyelitis/chronic fatigue syndrome which could explain the similarity in symptoms between these two conditions (21).

On another hand, Cecchetti et al. (22) found that COVID-19 patients showed interrelated cognitive, EEG and 3T T2-weighted structural MRI abnormalities 2 months after being discharged from the hospital. A UK Biobank longitudinal imaging study of 785 patients having been scanned by structural MRI (T1, T2 fluid attenuation inversion recovery (FLAIR) and susceptibility-weighted MRI) before, through indications for scans unrelated to COVID-19, and approximately 141 days after testing positive for COVID-19, showed statistically significant atrophy of gray matter in limbic cortical areas, directly linked to olfactory and gustatory systems 6 (23).

Another application for 3T T2-space structural MRI in post-COVID syndrome is demonstrated in Kandemirli et al.'s (24) study. Out of 23 patients with persistent COVID-19 olfactory dysfunction, nearly three-quarters had olfactory cleft opacification, a reduction in olfactory bulb volumes, change in bulb shape or signal anomalies (24). These MRI studies allowed for a better understanding of the damages inflicted on the olfactory pathway in the context of COVID-19 anosmia.

In a study conducted by Besteher et al. (25), increased gray matter volume clusters were found in post-COVID patients 8 months after their acute COVID-19 infection compared to controls using voxel-based morphometry (VBM). They used VBM with CAT12 toolbox, using T1-weighted images obtained from a 3T MRI to measure gray matter volumes. The clusters were found bilaterally in the frontal lobe, temporal lobe, insula, amygdala, hippocampus, basal ganglia, and thalamus. The volume of four of these clusters was found to decrease over time. The first cluster was in the anterior insula and some regions of the frontal lobe. The second cluster was scattered in the left temporal lobe. The third cluster was in the left post-central and precentral gyrus and finally, the fourth cluster was in parts of the temporal lobe, the right fusiform gyrus, the parahippocampal gyrus, and the hippocampus. It was hypothesized that the augmentation of the gray matter volumes was caused by compensatory processes or inflammation (25).

Hellgren et al.'s (26) observational cohort study compared the MRI imaging of post-COVID patients to the neuropsychological findings of these patients. 3T MRI (T2-FLAIR, T2-FSE, T1-FSE, T1-GRE, DWI and SWI) imaging was done on patients whose neurocognitive test results seemed concerning to researchers. The neurocognitive test performed was the Repeatable Battery for the Assessment of Neuropsychological Status (RBANS). Thirty-five post-COVID patients who had been hospitalized for their acute COVID-19 infection and who were discharged on average 6.5 months prior underwent a brain MRI which revealed subcortical white matter lesions in 71% of these patients. The lesions were primarily found in the frontal and parietal lobes near the gray-white matter junction. Compared to the patients with normal MRI results, the patients with white matter lesions had lower scores in the RBANS visuospatial index. It is worth noting that the functionality level of the patients with white matter lesions prior to their COVID-19 infection was lower compared to controls (26).

Campabadal et al. (27) using 3T structural MRI found a reduction in gray matter volume in the caudate nucleus, putamen, olfactory cortex, parahippocampal gyrus, straight gyrus, left amygdala, and the superior and inferior orbital gyri using voxel-based morphometry. A study by Yus et al. (28) using 3T structural MRI (3D-T1, T2-FLAIR) found no statistically significant findings in brain volumes.

### 3.5 Functional MRI

A functional MRI (fMRI) is able to estimate brain metabolism by using either blood-oxygen level dependant (BOLD) contrast or cerebral blood flow to measure activity in different regions of the brain. Resting-state fMRI (rsf-MRI) refers to an fMRI being performed while a patient is not performing any tasks (38). Limited studies have been published regarding functional MRI and its applicability in Neuro-PASC patients. Kiatkittikul et al. (29) showed abnormal brain connectivity on rsf-MRI which was concordant with 18 F-FDG-PET/CT findings. It was reported that one patient who had sensorineural hearing loss of the left ear as a consequence of COVID-19 showed hypometabolism of the left temporal region on 18 F-FDG PET/CT scans and anomalies in rsf-MRI in the same region (29). Another study using rsf-MRI, led by Churchill et al. (30), demonstrated that patients who are currently experiencing a larger number of Neuro-PASC symptoms tended to have altered connectivity between parietal, temporal, occipital and subcortical regions using BOLD rsf-MRI. Additionally, distinct patterns correlated to the intensity of PASC symptoms. They elude to the fact that this could be a useful tool to differentiate between Neuro-PASC and other non-COVID-related infections (30).

Arterial spin labeling (ASL) is an MRI technique that has been used to measure brain perfusion in Neuro-PASC patients. A study by Ajčević et al. (31) found that participants who still had cognitive impairment 2 to 10 months after the beginning of their acute COVID-19 infection had cerebral hypoperfusion patterns on ASL MRI imaging. The hypoperfusion was primarily found in the frontal cortex but was also present in the temporal and parietal cortex. These findings were more significant in the right hemisphere. They also found that overall, the cerebral blood flow was lower throughout the gray matter in Neuro-PASC patients compared to healthy controls (31). Yus et al. (28) found similar results in their ASL study on post-COVID patients where they found lower perfusion in the patient's orbital and medial frontal lobes.

### 3.6 Diffusion MRI

Diffusion MRI (dMRI) is an imaging modality that utilizes the diffusion of water as contrast. Diffusion tensor imaging (DTI) is a type of dMRI that creates a 3D construction of diffusion (39). Diffusivity measurements are indicators of tissue microstructure integrity. Douaud et al. (23) found a greater increase in diffusivity, an indicator of tissue damage, in areas functionally connected to the piriform cortex, olfactory tubercle and anterior olfactory nucleus, in patients who were infected with SARS-CoV-2 compared to healthy controls (23). Additionally, a study by Campabadal et al. (27) found that Neuro-PASC patients presenting with olfactory dysfunction had higher mean and radial diffusivity in certain white matter regions on DTI compared to post-acute COVID-19 patients without olfactory dysfunction. The areas of increased mean diffusivity were found in the genu of the corpus callosum, forceps minor, orbitofrontal white matter tracts, and anterior thalamic radiations. Regions with augmented radial diffusivity were found in the genu of the corpus callosum, anterior corona radiata, and uncinate fasciculus (27). In contrast, a study done by Yus et al. (28) found no statistically significant findings with DTI—in patients with Neuro-PASC.

### 3.7 Susceptibility-weighted imaging

Susceptibility-weighted imaging was also used to try to visualize the neurological sequela of SARS-CoV-2 infection. This imaging modality is an MRI technique that uses the patient's iron, calcium, and deoxygenated blood as a source of contrast for the image. This imaging modality is useful for detecting hemorrhages, traumatic brain injury, microvasculature, and neurodegenerative diseases (40). Hellgren et al.'s (26) study using SWI found mostly subcortical abnormalities near the gray-white matter junction in frontal and parietal lobes in 8 patients out of 35 (26).

In addition, Mishra et al. (32) found regions of abnormal susceptibility bilaterally in the brain stem, the gray-white matter junction and in the white matter of the frontal lobes in patients with post-COVID condition or patients who had recovered after COVID-19 infection. The abnormalities seen in the brainstem were primarily in the midbrain and the abnormalities in the frontal lobes were located in the uncinate fasciculus tract and the inferior frontal-occipital fasciculus tracts (32). However, it is important to note that this article has not been peer-reviewed. To our knowledge, this is the only published article where the main focus was the use of susceptibility-weighted imaging to find radiological markers in post-COVID patients.

## 4 Discussion

Overall, different cerebral imaging techniques have been evaluated to find a reliable and reproducible marker of the Neuro-PASC footprint. ^18^F-FDG-PET/CT and sMRI seem to be the most studied. SPECT scans, SWI, dMRI, fMRI, and ASL were not largely studied, however, deserve to be further investigated in the context of Neuro-PASC.

One current limitation of ^18^F-FDG-PET/CT and SPECT studies is that these studies' complexity, cost and radiation exposure limit the number of research cohorts. These types of imaging require prolonged periods in a scanner which is possibly not efficient when searching for a highly available, yet reliable method.

On the other hand, the use of ionizing radiation, as seen in ^18^F-FDG-PET/CT can only really be justified when there is high suspicion of important neurological complications. Thus, exposing patients to radiation when the benefits of such a technique remain uncertain can be perceived as questionable. Consent for such radiation exposure would be, as per usual, required.

While studies on ^18^F-FDG-PET/CT are ample, studies on SPECT are insufficient. Despite this, current literature suggests that because ^18^F-FDG-PET/CT has superior sensitivity, as well as better contrast and resolution than SPECT, currently, ^18^F-FDG-PET/CT imaging, is superior to SPECT. On the other hand, the cost of ^18^F-FDG PET/CT is significantly higher than SPECT and remains an important obstacle to its access in our healthcare system. Verger et al.'s (34) SPECT study also demonstrated that this imaging technique can be of particular interest when tracking the recovery from Neuro-PASC.

Although amyloid deposits were found in Ferrucci et al.'s study, they only conducted an amyloid PET scan on one patient. Further research is needed to conclude whether the amyloid PET scan is a useful imaging modality to visualize Neuro-PASC. A study from Sun et al. (41) showed significantly higher plasma levels of amyloid beta in participants after recovery from acute COVID-19 infection compared to controls. These results were present in participants whether they had perduring neurological symptoms or not (41). This also supports the need for further research.

With what pertains to structural MRI, findings varied depending on the strength of the MRI and the MRI technique. Structural MRI gave varying information on Neuro-PASC, such as localizing subacute infarcts and noting cortical atrophy. With a 3T MRI, some researchers were only able to localize areas of previous infarcts whilst others found atrophy of the gray matter in limbic cortical areas, a reduction in olfactory bulb volumes, or subcortical white matter lesions in the frontal and parietal lobes. On the other hand, with a 7T MRI, Thapaliya et al. found an increased volume of the brainstem in Neuro-PASC patients but the literature regarding 7T MRI in this population is limited. Clusters of increased gray matter volumes were found with voxel-based morphometry in the frontal lobe, temporal lobe, insula, amygdala, hippocampus, basal ganglia, and thalamus.

On the other hand, keeping in mind that studies are also quite limited, functional MRI shows concordant results to ^18^F-FDG-PET/CT, meaning findings of corresponding regions of hypometabolism and their symptomatic manifestations. ASL MRI found areas of hypoperfusion primarily in the frontal cortex but also in the temporal and parietal cortex, which concorded with SPECT findings.

Overall, most diffusion MRI studies showed changes in diffusivity in areas connected to the primary olfactory cortex. Increased diffusivity was also noted in the genu of the corpus callosum, orbitofrontal white matter tracts, anterior thalamic radiation, and forceps minor. The same study found a decrease in gray matter volumes in olfactory-related regions. These findings along with the increased mean and radial diffusivity could explain the persistent olfactory deficits found in patients.

Additionally, susceptibility-weighted imaging revealed low susceptibility mainly in the frontal lobes and brain stems, which is consistent with abnormalities detected by other imaging modalities. The anomalies found in the uncinate fasciculus tract could explain the memory and mental health problems that are often present in post-COVID patients. The orbitofrontal region of the brain has a major influence on smell and taste therefore the areas of abnormal susceptibility near the orbitofrontal gyri could explain the loss of smell and taste seen in post-COVID patients.

Combining these different neuro-imaging modalities, particularly ^18^F-FDG-PET/CT and sMRI, could be an interesting and rewarding approach in identifying neurological markers of Neuro-PASC. Starting with a structural imaging technique and then proceeding to functional or metabolic modalities could allow for a better grasp of the structural and metabolic long-term progression and ultimate impact of COVID-19 on the brain. A consideration to keep in mind is that pre-existing neurological conditions and neurocognitive disorders need to be considered when interpreting Neuro-PASC symptoms and imaging. Also, the reassurance of a proper diagnosis and management comforted many Neuro-PASC patients who had noticed psychological decline for a prolonged period after the initial infection, for no clear reason. Their memory, energy level and concentration were not restored post-infection, leaving them with great uncertainty regarding the cause of their psychological decline. The clear and visible perfusion defects on SPECT imaging provided great validation to patients living with these new post-infectious deficits and helped physicians make a positive diagnosis.

## 5 Conclusion

Overall, many studies have been conducted on the use of neuro-imaging in post-COVID syndrome patients with Neuro-PASC. Among these, ^18^F-FDG-PET/CT and sMRI seem to be the most studied and the most promising neuro-imaging modalities to identify Neuro-PASC markers. Further research needs to be conducted with regard to fMRI, dMRI, SPECT, amyloid PET, ASL MRI and susceptibility-weighted imaging. Although multiple structural MRI studies revealed significant abnormalities, these findings differed considerably in location within the brain and in type of abnormality from one study to another. These gaps in our knowledge do not allow us to confidently confirm the applicability of these imaging techniques in the detection, monitoring, and response to treatment. Future research should include homogenous and larger sample sizes and longitudinal data as much as possible to better identify and understand Neuro-PASC markers.

The cumulation of preliminary results of ^18^F-FDG-PET/CT showed that hypometabolism seen in reports could constitute a quantitative marker of cerebral damage of post-COVID syndrome. sMRI identified atrophy in white matter and changes in gray matter volumes, which could also be considered potential markers of Neuro-PASC. The possibility of having markers that allow a more accurate follow-up on brain damage, progression and response to therapy would not only allow us to better treat the increasing number of patients suffering from Neuro-PASC, but to better understand the pathophysiology of this cataclysmic disease.

## Author contributions

OC and LA are the main authors of the article, doing the literature review, building the article, and sending for revisions. MM, JS, and DM helped screen the search results, with the writing and editing of the manuscript. AE and JW provided feedback and corrections, based on their expertise, in neurosurgery, and diagnostic imaging, respectively. LC-W overlooked the project and provided feedback at every stage of the article. All authors contributed to the article and approved the submitted version.
